# Atypical Presentation of *IARS1* ‐Related Disorder: Expanding the Phenotype and Genotype

**DOI:** 10.1002/jmd2.70020

**Published:** 2025-05-12

**Authors:** Parith Wongkittichote, Kira E. Jonatzke, Benjamin T. Hyde, Lance W. Peterson, Mai He, Robert C. McKinstry, Anthony Antonellis, Marwan Shinawi

**Affiliations:** ^1^ Department of Pediatrics, Division of Genetics and Genomic Medicine Washington University School of Medicine St. Louis Missouri USA; ^2^ Department of Pediatrics, Faculty of Medicine Ramathibodi Hospital Mahidol University Bangkok Thailand; ^3^ Department of Human Genetics University of Michigan Ann Arbor Michigan USA; ^4^ Department of Pediatrics, Division of Rheumatology and Immunology Washington University School of Medicine St. Louis Missouri USA; ^5^ Department of Pathology and Immunology Washington University School of Medicine St. Louis Missouri USA; ^6^ Mallinckrodt Institute of Radiology Washington University School of Medicine St. Louis Missouri USA; ^7^ Department of Neurology University of Michigan Ann Arbor Michigan USA

**Keywords:** aminoacyl‐tRNA synthetase, aminoacyl‐tRNA synthetase deficiencies, growth retardation, impaired intellectual development, hypotonia, and hepatopathy, IARS1, immune dysregulation, isoleucine

## Abstract

Aminoacyl‐tRNA synthetases (ARSs) catalyze the formation of aminoacyl‐tRNA, which is required for protein translation. A growing number of cases are associated with ARS deficiencies. Pathogenic variants in *IARS1* (MIM# 600709), encoding cytoplasmic isoleucyl‐tRNA synthetase, have been associated with autosomal recessive growth retardation, impaired intellectual development, hypotonia, and hepatopathy (GRIDHH, OMIM# 617093). To date, 11 GRIDHH patients have been described. We identified a patient who presented with recurrent episodes of liver failure in the setting of preceding infection and neurocognitive delay, and who recently presented with a clinical picture consistent with chronic nonbacterial osteomyelitis/chronic recurrent multifocal osteomyelitis. Exome sequencing revealed that this patient is compound heterozygous for two *IARS1* variants: c.1193dupC;p.(Cys400LeufsTer32) and c.746A>G;p.(Asp249Gly). The frameshift variant is predicted to cause a loss of function, and functional analysis of the p.Asp249Gly variant was performed using baker's yeast. Wild‐type human *IARS1* has been shown to support robust yeast growth in the absence of the yeast ortholog, *ILS* , while human *IARS1* harboring p.Asp249Gly could not, indicating a loss‐of‐function effect. The proband was treated with isoleucine supplementation with subjective clinical improvement. Overall, we expand the molecular and clinical spectra of the *IARS1* ‐related disorder, highlight immune dysregulation as a possible novel manifestation of this disorder, and emphasize the utility of a yeast model system for functional studies. A larger cohort of patients is required to validate these observations and evaluate the efficacy of isoleucine supplementation for patients with GRIDHH.

1

**Summary**

- This case study expands the molecular and clinical spectrum of IARS1‐related GRIDHH disorder.
- Functional analysis using a yeast model demonstrated the loss‐of‐function effect of the novel p.Asp249Gly IARS1 variant, emphasizing the utility of yeast models for studying aminoacyl‐tRNA synthetase deficiencies.
- Immune dysregulation is identified as a potential novel manifestation of GRIDHH.
- A preliminary evidence suggesting isoleucine supplementation may improve clinical outcomes.

## 2 Introduction

Aminoacyl‐tRNA synthetases (ARSs) are a family of enzymes that catalyze the formation of aminoacyl‐tRNA, which is essential for protein translation [1]. In order to attach appropriate amino acids to tRNA, ARSs contain catalytic and tRNA anticodon recognition domains [2]. Similar to most ARSs, isoleucyl‐tRNA synthetase 1 (IARS1) and IARS2 are encoded by two separate genes that produce enzymes for the cytoplasm and mitochondria, respectively. These enzymes correspond to the distinct protein translation processes in each compartment [1]. Multiple ARSs have been associated with human diseases [1, 3]. Patients with recessive cytoplasmic ARS defects exhibit various phenotypes affecting multiple organ systems, while patients with mitochondrial ARS defects have clinical presentations overlapping with mitochondrial disorders [3, 4, 5, 6, 7, 8].

Pathogenic variants in the cytoplasmic (*IARS1*) and mitochondrial (*IARS2*) isoleucyl‐tRNA synthetase genes have been implicated in human inherited disease [9]. Defects in mitochondrial IARS2, encoded by *IARS2* (MIM# 612801), have been associated with autosomal recessive cataracts, growth hormone deficiency, sensory neuropathy, sensorineural hearing loss, and skeletal dysplasia (CAGSSS, OMIM# 616007) [10]. In contrast, defects in cytoplasmic IARS1, encoded by *IARS1* (MIM# 600709), cause autosomal recessive growth retardation, impaired intellectual development, hypotonia, and hepatopathy (GRIDHH, OMIM# 617093) [11]. To date, only 11 cases of GRIDHH have been reported [11, 12, 13, 14, 15, 16, 17]. Common findings in patients with GRIDHH include neonatal hepatitis and recurrent liver failure triggered by acute illness and fever, intrauterine growth retardation followed by postnatal growth restriction and failure to thrive, variable degrees of developmental delay/intellectual disability, abnormal brain imaging, and zinc deficiency. However, the emerging phenotype, variable clinical manifestations, and potential genotype–phenotype correlations are not well characterized.

Here, we report a patient with an *IARS1*‐related disorder who, in addition to the known presentation of recurrent liver failure and developmental delay, exhibited inflammatory osseous lesions most consistent with chronic nonbacterial osteomyelitis (CNO)/chronic recurrent multifocal osteomyelitis (CRMO) and severe hypercalcemia. Molecular analysis revealed compound heterozygous variants in *IARS1*, including one single base‐pair duplication and one missense variant. Functional studies of the missense variant in a yeast complementation model indicated a loss‐of‐function effect. Combined, these data support the pathogenicity of the identified *IARS1* alleles. To our knowledge, CNO/CRMO and hypercalcemia have not been previously reported in patients with GRIDHH, and we discuss potential mechanisms. In sum, our work expands the clinical phenotypes and molecular aspects of GRIDHH.

## 3 Materials and Methods

### 3.1 Case Description

The proband is a 7‐year‐old boy of Northern European ancestry. He was born full term via vaginal delivery to a 20‐year‐old mother and a 27‐year‐old father after an uncomplicated pregnancy except for a history of fetal growth restriction (FGR), which later resolved, and echogenic bowel. His birth weight was at 89th percentile and birth length was at 72nd percentile. He passed the newborn metabolic screen. His parents and paternal half‐brother were healthy. There is no history of autoimmune disease in the family.

**FIGURE 1:**
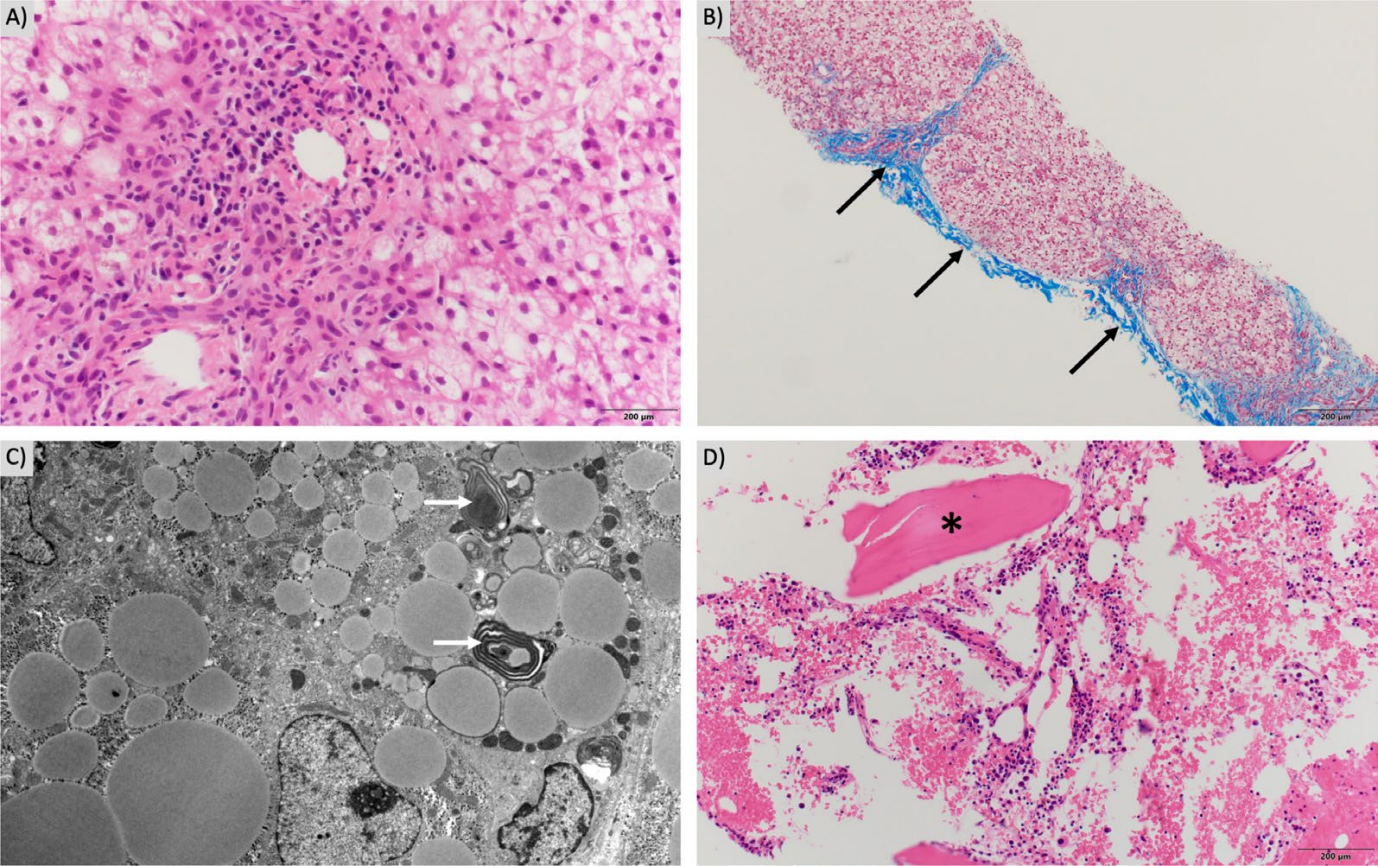
Histopathological studies of the proband. (A) Liver biopsies showed diffuse hepatocytic changes, with mixed pictures resembling cytoplasmic glycogen, some degree of hepatocytic ballooning, and macrovesicular changes. There are also ductal and ductular proliferations with pericholangitis. There are minimal to mild cholestasis. (B) Trichrome and reticulin stains of liver tissue demonstrate focal bridging fibrosis (dark arrows). (C) Electron microscope showed hepatic microvesicular steatosis, an increase in the number of phagolysosomes and myelin‐figures (light arrows). (D) Bone biopsies revealed reactive (shown in *) and lamellar bone with marrow elements. There is marrow edema and scattered as well as clustered plasma cells.

He was healthy until the age of 10 months when he presented with metabolic acidosis (increased anion gap), mild hyperammonemia (70–110 μmol/L, reference range (RR) < 50 μmol/L), elevated transaminases, coagulopathy, and hypoalbuminemia in the setting of influenza infection. This clinical picture was consistent with acute liver failure. His mildly elevated ammonia self‐resolved. Serum amino acid profile, carnitine levels, acylcarnitine profile, lactate/pyruvate, carbohydrate‐deficient transferrin, oxysterols, lysosomal enzyme screening studies (Thomas Jefferson University, Philadelphia, PA) were unremarkable. Urine organic acid analysis revealed a mild increase in adipic acid levels along with trace amounts of suberic and sebacic acids. The relative abundance of adipic acid in comparison to suberic and sebacic acids suggests a potential intake of dietary medium‐chain triglycerides (MCTs). Blood ketone level has not been obtained. His abdominal ultrasound showed diffusely increased hepatic echogenicity, small volume ascites, mild splenomegaly, and diffusely thickened and fluid‐filled colon loops. Respiratory chain complex enzymatic analysis of liver revealed an isolated decrease in Complex II–III activity (32% of controls) (Supporting Information Data S1). The mitochondrial DNA content (Baylor Genetics, Houston, TX) of liver tissue was also low (33% of matched controls for age and gender, Data S1); however, this finding could be nonspecific, and much lower levels are typically found in mitochondrial depletion syndromes. Liver biopsy revealed hepatopathy with focal bridging fibrosis, mild cholestasis, microvesicular and macrovesicular steatosis, ductal and ductular proliferation with pericholangitis (Figure 1A,B). Copper stain showed focal positivity. Electron microscopy showed hepatic microvesicular steatosis, an increase in the number of phagolysosomes and myelin figures (Figure 1C). Mitochondria were intact but had polymorphous unusual shapes and varied in number from cell to cell.

**FIGURE 2:**
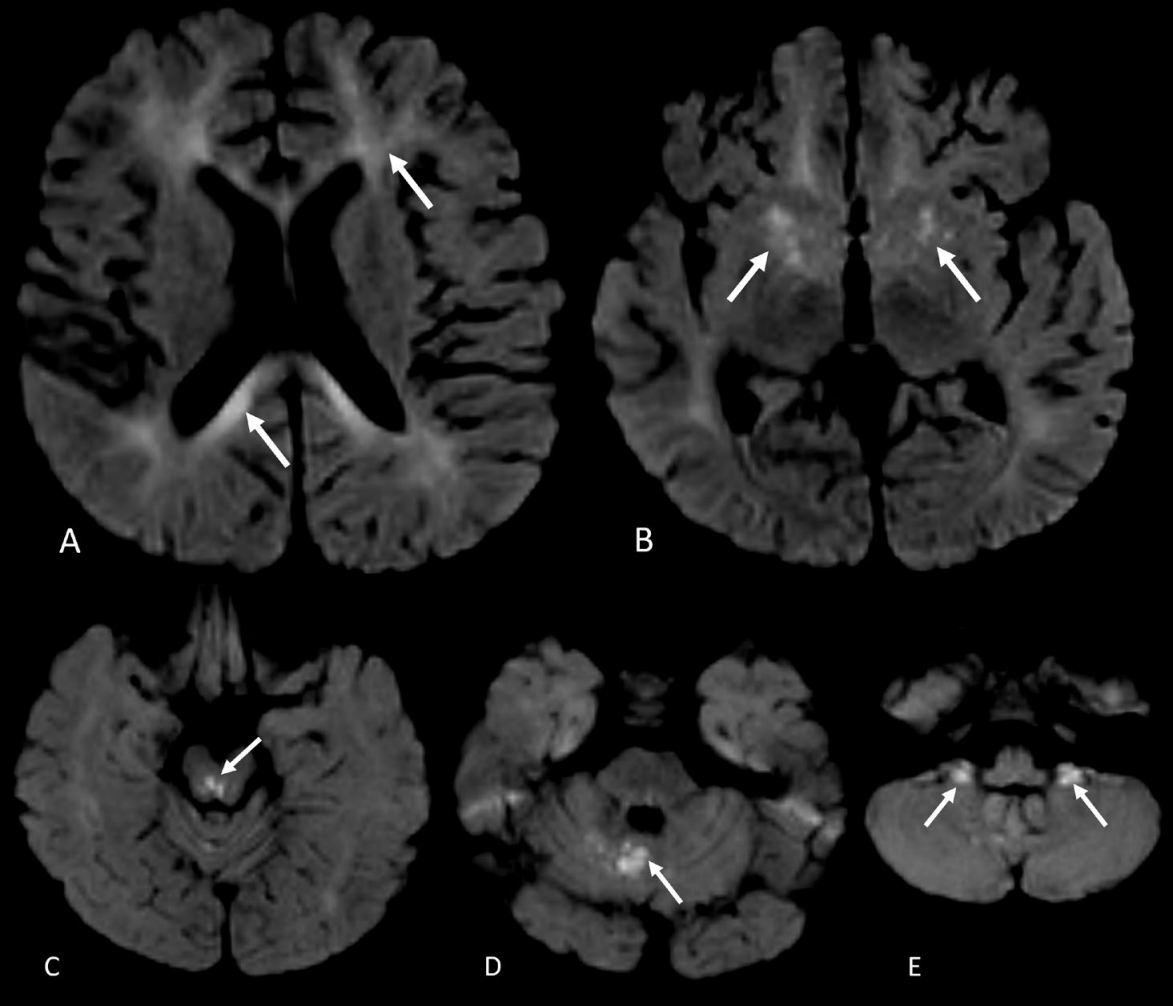
Neuroimaging of the proband. Restricted diffusion is noted in (A) deep white matter including corpus callosum, (B) lentiform nuclei, (C) midbrain tegmentum, (D) cerebellar vermis, and (E) cerebellar flocculi.

He presented again at the age of 3 years with altered mental status, viral symptoms, acholic stools, and a history of rapid weight loss. He was intubated for 3 days. Laboratory evaluation revealed elevated transaminases, gamma‐glutamyl transferase (GGT), mildly elevated ammonia (73 μmol/L), and low ceruloplasmin (11.2 mg/dL, RR 15–30 mg/dL). After the molecular results became available and showed two variants in *IARS1*, we obtained zinc levels that were normal (61 mcg/dL; RR: 29–115 mcg/dL). Because of prolonged encephalopathy, he had a brain magnetic resonance imaging (MRI) that showed patchy diffusion restriction with associated FLAIR hyperintensity in the lentiform nuclei, right occipital lobe, brainstem, and cerebellum as well as mild confluent diffusion restriction involving the cerebral white matter bilaterally and corpus callosum (Figure 2). Electroencephalogram (EEG) revealed several multifocal electrographic‐only seizures, for which he was started on levetiracetam. The background activity consisted of polymorphic delta activity and lacked normal waking and sleep patterns. Abdominal ultrasound showed a large liver with hyperechogenicity. He was also found to have subclinical hypothyroidism, which was treated with levothyroxine supplementation.

He was readmitted for 1 day at the age of 5 years for evaluation of altered mental status, fever, and decreased oral intake. His liver enzymes, lactate, and ammonia were normal. He was noted to have mild developmental delay. He could run and climb stairs but was described as clumsy. He also had fluctuations in his muscle strength and endurance per parental report, with significant muscle weakness toward the end of the day. He could use utensils but could not independently dress and undress. He also exhibited pronunciation difficulties.

He had an additional 5‐day admission when he was 6 years old for febrile illness due to respiratory syncytial virus (RSV) infection associated with mild elevation of liver enzymes. He received symptomatic treatment and dextrose‐containing fluids with complete recovery.

At the age of 7 years, he developed acute onset pain in bilateral lower legs, progressing to diffuse bone pain, and refusal to bear weight in the setting of adenoviral infection and 2 days of fever. Physical examination was notable for extreme tenderness over the anterior medial bilateral tibias, without any warmth, erythema, or swelling. He had pain with straightening his left leg, but preserved ranges of motion. Initial evaluation demonstrated C‐reactive protein (CRP 147 mg/L, RR < 10.0 mg/L), ferritin (515 ng/mL, RR 7–140 ng/mL), lactate dehydrogenase (LDH 416 units/L, RR 100–300 units/L), anemia (hemoglobin 8.8 g/dL, RR 11.5–15.5 g/dL), and thrombocytopenia (platelet count 100 × 10^3^/mm^3^, RR 140–400 × 10^3^/mm^3^). Plasma cytokine panel demonstrated significantly elevated IL‐6 (772 pg/mL RR < 8 pg/mL) and IL‐8 (149 pg/mL RR < 9 pg/mL). Despite this nonspecific evidence of systemic inflammation and bicytopenia, his triglycerides and fibrinogen were normal, liver enzymes remained at baseline, and he had no hepatosplenomegaly. He also had severe hypercalcemia (calcium 14.6 mg/dL, RR 8.5–10.3 mg/dL), coagulopathy (INR 1.99, RR 0.80–1.20), and acute kidney injury (creatine increased from baseline at 0.4 to 1.08 mg/dL, RR 0.20–0.80 mg/dL). MRI of bilateral lower legs revealed heterogeneous scattered abnormal T2 signals in both tibial shafts with a more prominent focus in the left proximal tibia and anterior soft tissue edema. Bone marrow biopsy showed reactive and lamellar bone with marrow edema and scattered plasma cell aggregates (Figure 1D), but no evidence of hemophagocytosis or malignancy, including the absence of CD1a staining to suggest Langerhans cell histiocytosis. He had negative infectious evaluations. His presumed diagnosis based on clinical course, radiological abnormalities, and pathological findings was CNO/CRMO. An obvious cause of his hypercalcemia has not been identified, as this is not a typical or reported complication of CNO/CRMO, 25‐hydroxyvitamin D level was normal, and 1,25‐dihydroxyvitamin D level was appropriately undetectable. Rapid whole‐genome sequencing did not identify any variants in genes implicated in monogenic inborn errors of immunity. Ultimately, he was started on intravenous methylprednisolone and a single infusion of zoledronic acid with resolution of diffuse bone pain, moderate improvement of focal tibial pain, and significant improvement in other laboratory values over just several days.

He was last seen at age 7 years 1 month. On physical examination, his weight was at the 61st percentile, height at 80th percentile, and occipitofrontal circumference (OFC) at the 23rd percentile. He had a broad forehead, slightly triangular face, droopy and deep‐set eyes, smooth philtrum, thin upper lip, bulbous nasal tip, and thin and sparse hair, especially in his temporal and occipital areas. His neurological exam was unremarkable.

Isoleucine supplementation was initiated as an off‐label therapeutic intervention based on clinical judgment and existing literature supporting its potential benefits in *IARS1*‐related disorder [18]. This treatment was not part of an experimental trial but rather implemented as standard clinical care. As such, Institutional Review Board (IRB) approval and formal informed consent for this intervention were not required. However, the proband's legal guardians were fully informed about the off‐label nature of the therapy, including its rationale, potential risks, and anticipated benefits, in accordance with ethical guidelines for clinical practice (see Ethics Statement). There have been no formal endpoints for this treatment, but the parents reported subjective improvement in the proband's endurance and level of daily activity since the isoleucine supplementation started. In addition, since the patient started isoleucine 12 months ago, he exhibited no major acute decompensation episodes.

### 3.2 Molecular Analysis

Clinical trio exome and trio genome sequencing in the proband was performed by GeneDx (Gaithersburg, MD). Genome sequencing was ordered when he presented with CNO/CRMO in an attempt to find another genetic etiology for his new inflammatory condition. The variants were confirmed by Sanger sequencing. Variant annotation and analysis were performed using the company's custom‐developed analysis tool. *In silico* analysis of the effect of missense variants was performed by various bioinformatic tools including combined annotation dependent depletion (CADD) [19], rare exome variant ensemble learner (Revel) [20], VARITY [21], sorting intolerant from tolerant (SIFT) [22], MutationTaster2 (MT) [23], and functional analysis through hidden markov models (FATHMM) [24]. Variant classification was performed according to the American College of Medical Genetics and Genomics (ACMG) recommendation [25].

### 3.3 Functional Analysis of the p.Asp249Gly *IARS1* Variant

**FIGURE 3:**
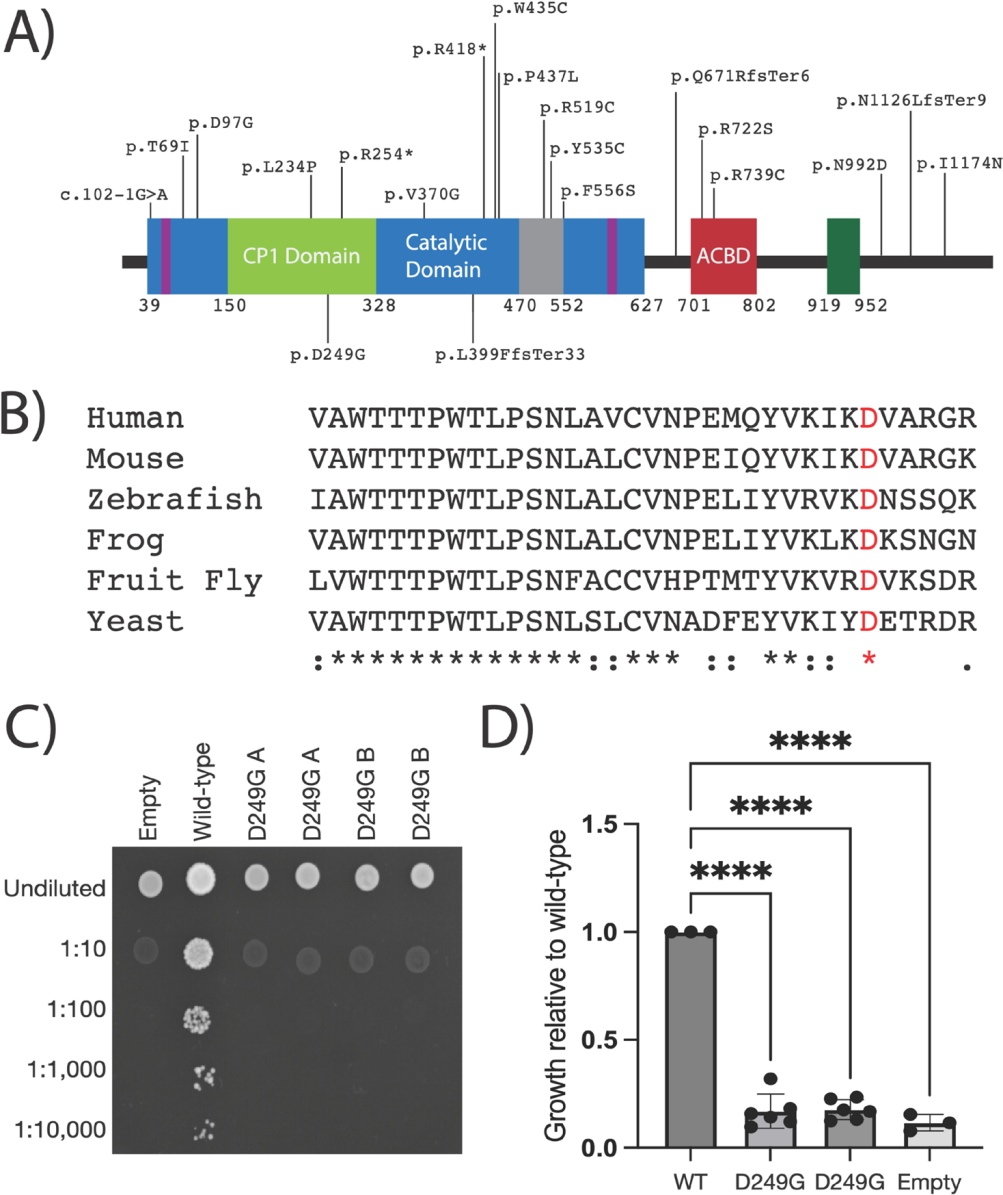
Characterization of pathogenic *IARS1* variants. (A) A cartoon of the IARS1 protein is shown with major functional domains indicated along with disease‐associated *IARS1* variants previously described and those identified in this study. The catalytic domain (blue), CP1 domain (light green), and anticodon binding domain (ACBD, red) were determined based on conservation comparisons to *Mycobacterium tuberculosis* isoleucyl‐tRNA synthetase [26]. The ATP‐binding sites (purple), CP2 domain (gray), and Zinc‐binding domain (dark green) were determined based on conservation comparisons to yeast [27]. The variants identified in this study are shown below the protein while previously identified variants are shown above the protein. Numbers along the bottom of the protein indicate amino‐acid positions. (B) A multiple‐species protein sequence alignment is shown for IARS1 residues surrounding the p.Asp249 residue. Species are indicated along the left, conservation is indicated along the bottom, and the p.Asp249 residue is indicated in red. (**C**) Yeast lacking endogenous *ILS* (the yeast ortholog of *IARS1*) and maintained by a *URA3*‐bearing plasmid bearing wild‐type *ILS* were transformed with plasmids containing wild‐type *IARS1*, p.Asp249Gly *IARS1* (noted as “D249G”), or no insert (“Empty”). Resulting cultures were plated undiluted or diluted (1:10, 1:100, 1:1000, or 1:10 000) on media containing 5‐FOA. Two independently generated mutant constructs (“A” and” “B”) were generated. (D) Quantification of yeast growth at day three across three independent trials. All data are normalized to growth associated with wild‐type *IARS1*. Constructs are indicated along the bottom and “****” indicates *p* < 0.0001.

Yeast complementation assays were performed as previously described [13]. The p.Asp249Gly *IARS1* variant was modeled in the human wild‐type *IARS1* open reading frame. Mutagenesis was performed using the Quickchange II XL Site‐Directed Mutagenesis Kit (Agilent) and primers designed to introduce the p.Asp249Gly *IARS1* variant into wild‐type *IARS1* previously cloned into the pDONR221 vector (Thermo Fisher Scientific). Full plasmid sequencing verified this change and the absence of any unwanted mutations. Two independently generated p.Asp249Gly *IARS1* pDONR221 constructs (“A” and “B” in Figure 3C,D) and none wild‐type *IARS1* pDONR221 construct were cloned into the pYY1 expression vector [28], which has a *LEU2* gene, using Gateway cloning technology (Thermo Fisher Scientific) before being transformed into *Escherichia coli*. Plasmid DNA recombination was verified via *Bsr*GI digest. Each plasmid was independently transformed into yeast harboring a deletion of *ILS* (the yeast *IARS1* ortholog) with viability supported by a *URA3*‐bearing vector with a wild‐type copy of *ILS*. Colonies were grown on media lacking leucine and uracil to select for successfully transformed yeast. Colonies were selected and grown in 2 mL of liquid media without leucine and uracil for 48 h at 30°C and shaking at 275 rpm. One milliliterof the culture was spun down and the pelleted yeast cells were resuspended in 50 μL of UltraPure RNase/DNase‐free water. Undiluted cultures and diluted cultures (1:10, 1:100, 1:1000, and 1:10 000) were spotted on 0.1% 5‐FOA media, which selects for yeast that have spontaneously lost the *URA3*‐bearing vector [29]. Viability was assessed after 3 days of incubation at 30°C and colony growth was quantified on solid media using ImageJ [30]. This experiment was repeated five times using five independent colony sets from three independent transformations.

## 4 Results

### 4.1 Exome Sequencing Identified Variants in *IARS1*

Clinical trio exome sequencing identified two *IARS1* (NM_002161.5) variants in the heterozygous state: a maternally inherited frameshift variant designated as c.1193dupC (p.Cys400LeufsTer32) and a paternally inherited missense variant designated as c.746A>G (p.Asp249Gly). The c.1193dupC variant was reported in gnomAD (frequency 1/248 968) while the p.Asp249Gly was not reported in the same database. The p.Asp249 residue maps to the CP1 domain (predicted to play a role in enzyme fidelity; Figure 3A) within the catalytic core and is highly conserved among many species, including human and yeast (Figure 3B). The p.Cys400LeufsTer32 *IARS1* variant maps to the middle of the catalytic domain and is predicted to ablate most of the catalytic domain and all of the anticodon binding domain (Figure 3A). In silico analysis of the missense variant yielded conflicting results (Table 1). When the proband presented with CNO/CRMO and unexplained hypercalcemia, he underwent clinical trio rapid whole‐genome sequencing, which did not identify additional variants, including variants in genes implicated in monogenic inborn errors of immunity and CNO/CRMO, including *IL1R1*, *IL1RN*, *LPIN2*, *FBLIM1*, and *NLRP3* [31].

**TABLE 1:** In silico prediction and ACMG classification of the missense variant c.746A>G;p.(Asp249Gly).

Tool	Score/classification
CADD	25.1
Revel	Uncertain (0.62)
Varity	Deleterious (0.82)
SIFT	Uncertain (0)
MT	Deleterious (1)
FATHMM	Uncertain (858801.02)
ACMG criteria	PS3, PM2, PM3, PP4
Interpretation	Likely pathogenic

### 4.2 Yeast Complementation Study

We have successfully employed yeast complementation assays to study the functional consequences of numerous pathogenic ARS variants, including those in *IARS1* [13, 32]. Importantly, pathogenic ARS variants that have shown loss‐of‐function effects in yeast in vivo assays have shown loss‐of‐function effects in enzyme kinetic assays [32], supporting the utility of yeast complementation assays in studying ARS variants. Wild‐type human *IARS1* has been shown to support robust yeast growth [13] in the absence of the yeast ortholog, *ILS*. This allows us to compare the ability of wild‐type *IARS1* versus p.Asp249Gly *IARS1* to support yeast cell growth. Two independent constructs to express p.Asp249Gly *IARS1* (“A” and “B” in Figure 3C,D), one construct to express wild‐type *IARS1*, and one construct with no *IARS1* insert (“Empty” in Figure 3C,D) were independently transformed into a yeast strain harboring a deletion of the endogenous yeast *IARS1* (*ILS*) and carrying a maintenance vector that expresses *URA3* and a wild‐type copy of *ILS*. After transformation, yeast strains were plated on 5‐FOA, which selects for cells that have spontaneously lost the *URA3*‐bearing maintenance vector [29]. The plasmid with no *IARS1* insert did not support yeast growth (Figure 3C,D) confirming that isoleucyl‐tRNA synthetase is an essential gene in yeast. The wild‐type *IARS1* construct supported robust yeast growth (Figure 3C,D) confirming that human *IARS1* can complement the deletion of yeast *ILS*. The p.Asp249Gly *IARS1* constructs were unable to support any yeast growth (Figure 3C,D) indicating that this is a loss‐of‐function allele, consistent with pathogenicity in the observed recessive phenotype. Finally, we attempted to rescue the loss‐of‐function effect of p.Asp249Gly *IARS1* by supplementing yeast cells with a range of isoleucine concentrations up to 250 mg/mL; however, we did not see any improvement in yeast cell growth with any concentration of isoleucine (Figure S1).

## 5 Discussion

To date, there are 12 GRIDHH patients (including the proband in this report) from 11 families that have been reported in the literature (Table 2) [11, 12, 13, 14, 15, 17]. Variable degrees of developmental delay and/or intellectual disabilities have been reported in all patients except one (11/12) while 44% (4/9) had abnormal brain MRI, mostly affecting the white matter of the brain. Liver involvement is present in almost all reported patients (10/12) with elevated transaminases (9/12) and liver synthetic dysfunction (8/12) being common findings. Hepatomegaly has been reported in 50% (6/12). Among the patients who underwent liver biopsy, hepatic steatosis is the most common histological finding, followed by cholestasis. Zinc deficiency was found in 50% (6/12). FGR and growth failure, which were thought of as some of the cardinal features of this condition, are not present in the proband reported here and another patient [16]. This may suggest that *IARS1*‐related disorder would be a more appropriate nomenclature for this condition than GRIDHH.

**TABLE 2:** Clinical and molecular characteristics of known patients with *IARS1*‐related disorders.

	#65269 (Kopajtich et al. [11])	#85880 (Kopajtich et al. [11])	#83921 (Kopajtich et al. [11])	Orenstein et al. [13]	Smigiel et al. [14]	P1 Fuchs et al. [3]	P2 Fuchs et al. [3]	Fagbemi et al. [16]	Guojie et al. (2021) [17]	Zou et al. [15]	Jiang et al. [12]	This study
Genetic variants	c.1252C>T (p.Arg418Ter) c.3521T>A (p.Ile1174Asn)	c.760C>T (p.Arg254Ter) c.1310C>T (p.Pro437Leu)	c.1109 T>G (p.Val370Gly) c.2974A>G (p.Asn992Asp)	c.2215C>T, (p.Arg739Cys) c.1667T>C, (p.Phe556Ser)	c.2011delC (p.Gln671fs), c.206C>T (p.Thr69Ile)	c.1305G>C (p.Trp435Cys), c.3377dup (p.Asn1126fs)	c.1305G>C (p.Trp435Cys), c.3377dup (p.Asn1126fs)	c.290A>G (p.Asp97Gly)	c.1604A>G (p.Tyr535Cys)	c.701T>C (p.Leu234Pro), c.1555C>T (p.Arg519Cys)	c.120‐1G>A, c.2164C>A (p.Arg722Ser)	c.1193dupC (p.Cys400fs), c.746A>G (p.Asp249Gly).
Sex	M	F	M	M	M	F	M	M	M	F	F	M
Age at last visit	18.7 years	19 years	3 years	4 years	7 years	4 months (died)	5 years	9 years	6 years	19 months (died)	17 months	7 years
IUGR	+	+	+	+	+	+	+	N/A	N/A	+	+	−
Oligohydramnios	−	−	−	+	−	−	−	N/A	−	+	−	−
Growth retardation	+	+	+	+	+	+	+	−	+	+	+	−
ID/DD	Moderate to severe	Mild to moderate	Moderate	Mild to moderate	+	+	+	−	+	Moderate to severe	+	+
Regression	−	−	−	˗	−	−	−	−	−	−	+	−
Microcephaly	+	˗	+	+	−	+	−	+	−	+	+	−
Hypotonia	+	−	+	−	+	+	+	−	+	+	+	−
Movement Disorder	Bilateral spasticity	−	−	−	−	−	−	−	N/A	−	−	−
Brain MRI	White matter changes consistent with hypomyelination	Normal	Normal	N/A	Normal	N/A	Sparse white matter with neuronal damage in the basal ganglia and thalamus on autopsy	N/A	Normal	Unclear boundary between gray matter and white matter, slightly widened and deepened sulci and cistern	Normal	Patchy diffusion restriction with associated FLAIR hyperintensity in the lentiform nuclei, right occipital lobe, brainstem, and cerebellum
Dysmorphic features	N/A	N/A	Chubby cheeks	Round face, full cheeks, abnormal fat distribution	Baldness, delicate hair, thin and long eyelashes, prominent cheeks, flat philtrum	N/A	N/A	N/A	Peculiar facial features	Chubby and flabby face	A prominent left ear, peculiar facial features	Broad forehead and slightly triangular face
Joint hyperlaxity	−	−	−	+	−	−	−	N/A	−	+	−	−
Hyperelastic Skin	−	−	−	+	−	−	−	N/A	−	+	−	−
Elevated AST/ALT	−	+	+	+	+	+	+	+	−	+	+	+
Liver synthetic dysfunction	+	−	+	+	−	+	+	+	−	+	+	+
Hepatomegaly	−	−	−	−	+	+	+	+	−	+	−	+
Liver biopsy	N/A	Steatosis and portal‐tract fibrosis	Steatosis, fibrosis, cholestasis	Cholestasis and fibrosis	Steatosis	Micro‐ and macrovesicular steatosis	N/A	Hydropic degeneration of hepatocytes	N/A	N/A	N/A	Fibrosis, mild cholestasis, steatosis, pericholangitis
Hearing loss	−	+	−	−	−	−	−	−	−	−	−	−
Diabetes mellitus	−	+	−	−	−	−	−	−	−	−	−	−
Recurrent infection	+	−	+	−	+	−	−	−	+	+	+	+
Anemia	N/A	N/A	N/A	N/A	N/A	+	+	+	−	N/A	−	+
Thrombocytopenia—thronbocytosis	N/A	N/A	N/A	N/A	N/A	+	+		N/A	N/A	−	+
Elevated ESR and/or CRP	N/A	N/A	N/A	N/A	N/A	+	+	+	N/A	N/A	N/A	+
Elevated ferritin	N/A	N/A	N/A	N/A	N/A	+	+	N/A	N/A	N/A	N/A	+
Immune dysregulation	−	−	−	−	−	−	−	Early‐onset IBD	−	−	−	CNO/CRMO
Poor feeding	+	−	+	−	+	+	+	+	−	+	+	−
Zinc deficiency	+	+	+	−	+	−	−	−	+	−	−	−
OXPHOS activities	Decreased Complex I and IV in muscle	Decreased Complex I in fibroblasts	Decreased Complex I in liver	N/A	N/A	N/A	N/A	N/A	N/A	N/A	N/A	Decrease in Complex II–III activity in fibroblast

Hypotonia and microcephaly are relatively common among patients with GRIDHH, with a frequency of 67% (8/12) and 58% (7/12), respectively. Dysmorphic features have been observed in patients with GRIDHH, with a chubby face/full cheeks being the most common characteristic; however, other dysmorphic features are nonspecific, which limits the usefulness of these features for suspecting diagnosis.

Mitochondrial electron transport complex analysis is abnormal in all patients who underwent the testing (4/4, Table 2) [11], although the patterns are not specific. These abnormalities may reflect secondary mitochondrial dysfunction in patients with GRIDHH. In fact, mitochondrial dysfunction was demonstrated in *IARS1*‐deficient HepG2 cells and in a mouse model [33]. Mice harboring mutant *IARS1* were found to have decreased expression of *Nme4*, which encodes mitochondrial nucleoside diphosphate kinase, a protein located in the intermembrane space. NME4 is predicted to stabilize mitochondrial membranes via an interaction with anion phospholipids [33, 34]. The defects in NME4 cause abnormal mitochondrial morphology similar to those found in mitochondrial fusion defects [34]. Therefore, loss of NME4 expression may be one of the major mechanisms for secondary mitochondrial dysfunction in the patients with GRIDHH.

Immunologic abnormalities have been reported among GRIDHH patients with frequent infections as the most common feature (63%, 7/11). Recurrent infections have also been reported in Japanese Black cows affected with *IARS1*‐related perinatal weak calf syndrome [35]. Recently, immune dysregulation has also been reported in a patient with GRIDHH in the form of refractory early‐onset inflammatory bowel disease (IBD) [16]. The proband in this report exhibited a history of recurrent infections and inflammatory osseous lesions. Biopsy of the main lesion revealed the features overlapping with CNO/CRMO; however, hypercalcemia, which is not typically associated with CNO/CRMO, was also present. With these findings, it is likely that *IARS1* deficiency not only leads to immunodeficiency but also to autoinflammatory phenotypes. The mice harboring mutant *Iars1* have been shown to have abnormal expression of multiple immune system genes, for example, *Jak3*, *Il17ra*, and *Irf8* [33]. It is possible that dysregulation of immune genes may lead to abnormal immune phenotypes, but the mechanism of the immune abnormalities in GRIDHH has yet to be revealed, and larger cohorts of the patients are needed to establish the immunological phenotype of GRIDHH.

From a molecular genetic perspective, most patients with *IARS1*‐related phenotypes are compound heterozygous for two variants, with the exception of two patients who are homozygous for a single variant. The majority of reported variants are private to each individual. Among the 20 disease‐associated variants identified, missense variants are the most prevalent, comprising 70% (14/20), while the remaining variants include 2 nonsense variants (10%), 1 splice‐site variant (5%), and 3 frameshift deletions or duplications (15%). The variants are dispersed throughout the gene without any obvious hotspots (Figure 3A). These findings complicate the molecular diagnosis of the disease.

The c.1193dupC variant in the proband is predicted to lead to a frameshift and protein truncation; therefore, it is likely to cause a null allele due to nonsense‐mediated decay (NMD) or a non‐functional truncated protein (i.e., one with half of the catalytic domain and a deleted tRNA recognition domain). To establish the pathogenicity of ARS missense variants, studies using model organisms are conducted to assess the impact of these variants on gene function. The failure of human p.Asp249Gly *IARS1* to support yeast growth suggests a loss‐of‐function mechanism for this variant, similar to other tested pathogenic ARS alleles [32]. A major advantage of this system is the ability to employ human *IARS1* and to compare the wild‐type to the mutant human gene. However, there are important limitations to the yeast model, which impact interpretations. For example, while a human ARS can often rescue deletion of the yeast ARS, the human gene often supports less robust yeast growth compared to the yeast gene. This tempered rescue may result in pathogenic human mutations having a more severe effect compared to the effect in human tissues; this may also explain the lack of rescue in yeast upon treatment with high concentrations of isoleucine. Indeed, based on the nature of the frameshift allele described here, we predict that p.Asp249Gly *IARS1* has some residual function to account for the viability of the patient. Despite this limitation, yeast still presents an informative model system to compare the function of wild‐type and mutant human ARSs.

Treatment of GRIDHH was previously based on symptomatic treatment with zinc supplementation if zinc deficiency is present. A recent study revealed that supplementation of cognate amino acids to different ARS deficiencies leads to significant improvement in the patients [18]. To test this, our proband was treated with isoleucine 60 mg/kg/day about 6 months prior to his last visit with improvement in his muscle weakness and endurance by parental report, which is consistent with the improvement in feeding, growth and development, pulmonary involvement, and biochemical parameters in previously reported GRIDHH patients supplemented with isoleucine [18]. Thus, our study supports the supplementation of isoleucine in GRIDHH patients; however, larger cohorts are needed to establish the safety and efficacy of isoleucine supplementation.

Here, we describe an additional case of GRIDHH and expand the phenotypic and molecular spectra of the disease. Our study highlights potential immunologic dysregulation as a significant complication of GRIDHH. We also emphasize the utility of the yeast models for studying GRIDHH and other ARS deficiencies. While isoleucine supplementation can potentially improve the clinical course of GRIDHH, further clinical studies are needed to confirm our observations.

## Author Contributions

P.W. and M.S. designed and conceptualized the study. P.W., L.W.P., M.H., R.C.M., and M.S. performed clinical analysis of the patients. P.W., A.A., and M.S. performed variant analysis. P.W. drafted the manuscript. K.E.J., B.T.H., and A.A. performed functional study in the yeast model system. M.S. obtained consents. M.S. and A.A. supervised the study. All authors were involved with revising the manuscript.

## Ethics Statement

No interventions performed and no biological specimens collected from participant. The guardian of the patient signed a consent form for publication approved by Washington University IRB (Media Authorization for the Use and Disclosure of Protected Health Information).

## Consent

Consent was obtained from the patient's family for publication of this report.

## Conflicts of Interest

The authors declare no conflicts of interest.

## Supporting information


**Data S1.** Supporting Information.

## Data Availability

Yeast complementation reagents are available upon request.

## References

[1] A. Antonellis and E. D. Green , “The Role of Aminoacyl‐tRNA Synthetases in Genetic Diseases,” Annual Review of Genomics and Human Genetics 9 (2008): 87–107, 10.1146/annurev.genom.9.081307.164204. 18767960

[2] V. Rajendran , P. Kalita , H. Shukla , A. Kumar , and T. Tripathi , “Aminoacyl‐tRNA Synthetases: Structure, Function, and Drug Discovery,” International Journal of Biological Macromolecules 111 (2018): 400–414, 10.1016/j.ijbiomac.2017.12.157. 29305884

[3] S. A. Fuchs , I. F. Schene , G. Kok , et al., “Aminoacyl‐tRNA Synthetase Deficiencies in Search of Common Themes,” Genetics in Medicine 21 (2019): 319–330, 10.1038/s41436-018-0048-y. 29875423 PMC7091658

[4] M. Sissler , L. E. Gonzalez‐Serrano , and E. Westhof , “Recent Advances in Mitochondrial Aminoacyl‐tRNA Synthetases and Disease,” Trends in Molecular Medicine 23 (2017): 693–708, 10.1016/j.molmed.2017.06.002. 28716624

[5] L. Jiang , J. Jones , and X.‐L. Yang , “Human Diseases Linked to Cytoplasmic Aminoacyl‐tRNA Synthetases,” Enzyme 48 (2020): 277–319, 10.1016/bs.enz.2020.06.009. 33837707

[6] M. E. Kuo and A. Antonellis , “Ubiquitously Expressed Proteins and Restricted Phenotypes: Exploring Cell‐Specific Sensitivities to Impaired tRNA Charging,” Trends in Genetics 36 (2020): 105–117, 10.1016/j.tig.2019.11.007. 31839378 PMC6980692

[7] R. Meyer‐Schuman and A. Antonellis , “Emerging Mechanisms of Aminoacyl‐tRNA Synthetase Mutations in Recessive and Dominant Human Disease,” Human Molecular Genetics 26 (2017): R114–R127, 10.1093/hmg/ddx231. 28633377 PMC5886470

[8] C. Del Greco and A. Antonellis , “The Role of Nuclear‐Encoded Mitochondrial tRNA Charging Enzymes in Human Inherited Disease,” Genes 13 (2022): 2319, 10.3390/genes13122319. 36553587 PMC9777667

[9] K. Shiba , N. Suzuki , K. Shigesada , Y. Namba , P. Schimmel , and T. Noda , “Human Cytoplasmic Isoleucyl‐tRNA Synthetase: Selective Divergence of the Anticodon‐Binding Domain and Acquisition of a New Structural Unit,” Proceedings of the National Academy of Sciences of the United States of America 91 (1994): 7435–7439, 10.1073/pnas.91.16.7435. 8052601 PMC44415

[10] J. Schwartzentruber , D. Buhas , J. Majewski , et al., “Mutation in the Nuclear‐Encoded Mitochondrial Isoleucyl‐tRNA Synthetase IARS2 in Patients With Cataracts, Growth Hormone Deficiency With Short Stature, Partial Sensorineural Deafness, and Peripheral Neuropathy or With Leigh Syndrome,” Human Mutation 35, no. 11 (2014): 1285–1289, 10.1002/humu.22629. 25130867

[11] R. Kopajtich , K. Murayama , A. R. Janecke , et al., “Biallelic IARS Mutations Cause Growth Retardation With Prenatal Onset, Intellectual Disability, Muscular Hypotonia, and Infantile Hepatopathy,” American Journal of Human Genetics 99 (2016): 414–422, 10.1016/j.ajhg.2016.05.027. 27426735 PMC4974065

[12] J. Jiang , Y. Feng , Q. Tang , et al., “Novel IARS1 Variants Cause Syndromic Developmental Disorder With Epilepsy in a Chinese Patient and the Literature Review,” Molecular Genetics & Genomic Medicine 12 (2024): e2326, 10.1002/mgg3.2326. 38014478 PMC10767687

[13] N. Orenstein , K. Weiss , S. N. Oprescu , et al., “Bi‐Allelic IARS Mutations in a Child With Intra‐Uterine Growth Retardation, Neonatal Cholestasis, and Mild Developmental Delay,” Clinical Genetics 91 (2017): 913–917, 10.1111/cge.12930. 27891590 PMC5639925

[14] R. Smigiel , M. Biela , A. Biernacka , et al., “New Evidence for Association of Recessive IARS Gene Mutations With Hepatopathy, Hypotonia, Intellectual Disability and Growth Retardation,” Clinical Genetics 92 (2017): 671–673, 10.1111/cge.13080. 29052218

[15] T. T. Zou , H. Q. Sun , Y. Zhu , et al., “Compound Heterozygous Variations in IARS1 Cause Recurrent Liver Failure and Growth Retardation in a Chinese Patient: A Case Report,” BMC Pediatrics 22 (2022): 329, 10.1186/s12887-022-03371-6. 35668413 PMC9172121

[16] A. Fagbemi , W. G. Newman , S. G. Tangye , S. M. Hughes , E. Cheesman , and P. D. Arkwright , “Refractory Very Early‐Onset Inflammatory Bowel Disease Associated With Cytosolic Isoleucyl‐tRNA Synthetase Deficiency: A Case Report,” World Journal of Gastroenterology 26 (2020): 1841–1846, 10.3748/wjg.v26.i15.1841. 32351297 PMC7183863

[17] W. B. R. Guojie , R. Y. Baoerhan , D. L. Julaiti , and M. R. Maimaiti , “A Case of GRIDHH due to Mutations in the IARS Gene,” Chinese Journal of Birth Health & Heredity 29 (2021): 94–95.

[18] G. Kok , L. Tseng , I. F. Schene , et al., “Treatment of ARS Deficiencies With Specific Amino Acids,” Genetics in Medicine 23 (2021): 2202–2207, 10.1038/s41436-021-01249-z. 34194004 PMC8244667

[19] P. Rentzsch , D. Witten , G. M. Cooper , J. Shendure , and M. Kircher , “CADD: Predicting the Deleteriousness of Variants Throughout the Human Genome,” Nucleic Acids Research 47 (2019): D886–D894, 10.1093/nar/gky1016. 30371827 PMC6323892

[20] N. M. Ioannidis , J. H. Rothstein , V. Pejaver , et al., “REVEL: An Ensemble Method for Predicting the Pathogenicity of Rare Missense Variants,” American Journal of Human Genetics 99 (2016): 877–885, 10.1016/j.ajhg.2016.08.016. 27666373 PMC5065685

[21] Y. Wu , R. Li , S. Sun , J. Weile , and F. P. Roth , “Improved Pathogenicity Prediction for Rare Human Missense Variants,” American Journal of Human Genetics 108 (2021): 1891–1906, 10.1016/j.ajhg.2021.08.012. 34551312 PMC8546039

[22] R. Vaser , S. Adusumalli , S. N. Leng , M. Sikic , and P. C. Ng , “SIFT Missense Predictions for Genomes,” Nature Protocols 11 (2016): 1–9, 10.1038/nprot.2015.123. 26633127

[23] J. M. Schwarz , D. N. Cooper , M. Schuelke , and D. Seelow , “MutationTaster2: Mutation Prediction for the Deep‐Sequencing Age,” Nature Methods 11 (2014): 361–362, 10.1038/nmeth.2890. 24681721

[24] H. A. Shihab , J. Gough , D. N. Cooper , et al., “Predicting the Functional, Molecular, and Phenotypic Consequences of Amino Acid Substitutions Using Hidden Markov Models,” Human Mutation 34 (2013): 57–65, 10.1002/humu.22225. 23033316 PMC3558800

[25] S. Richards , N. Aziz , S. Bale , et al., “Standards and Guidelines for the Interpretation of Sequence Variants: A Joint Consensus Recommendation of the American College of Medical Genetics and Genomics and the Association for Molecular Pathology,” Genetics in Medicine 17 (2015): 405–424, 10.1038/gim.2015.30. 25741868 PMC4544753

[26] K. Shiba and P. Schimmel , “Functional Assembly of a Randomly Cleaved Protein,” Proceedings of the National Academy of Sciences of the United States of America 89 (1992): 1880–1884, 10.1073/pnas.89.5.1880. 1542687 PMC48557

[27] M. Sassanfar , J. E. Kranz , P. Gallant , P. Schimmel , and K. Shiba , “A Eubacterial *Mycobacterium tuberculosis* tRNA Synthetase Is Eukaryote‐Like and Resistant to a Eubacterial‐Specific Antisynthetase Drug,” Biochemistry 35 (1996): 9995–10003, 10.1021/bi9603027. 8756461

[28] C. I. Chien , Y. W. Chen , Y. H. Wu , C. Y. Chang , T. L. Wang , and C. C. Wang , “Functional Substitution of a Eukaryotic Glycyl‐tRNA Synthetase With an Evolutionarily Unrelated Bacterial Cognate Enzyme,” PLoS One 9 (2014): e94659, 10.1371/journal.pone.0094659. 24743154 PMC3990555

[29] J. D. Boeke , J. Trueheart , G. Natsoulis , and G. R. Fink , “5‐Fluoroorotic Acid as a Selective Agent in Yeast Molecular Genetics,” Methods in Enzymology 154 (1987): 164–175, 10.1016/0076-6879(87)54076-9. 3323810

[30] C. A. Schneider , W. S. Rasband , and K. W. Eliceiri , “NIH Image to ImageJ: 25 Years of Image Analysis,” Nature Methods 9 (2012): 671–675, 10.1038/nmeth.2089. 22930834 PMC5554542

[31] A. J. Cox and P. J. Ferguson , “Update on the Genetics of Nonbacterial Osteomyelitis in Humans,” Current Opinion in Rheumatology 30 (2018): 521–525, 10.1097/BOR.0000000000000530. 29912021

[32] S. N. Oprescu , L. B. Griffin , A. A. Beg , and A. Antonellis , “Predicting the Pathogenicity of Aminoacyl‐tRNA Synthetase Mutations,” Methods 113 (2017): 139–151, 10.1016/j.ymeth.2016.11.013. 27876679 PMC5253330

[33] M. Watanabe , K. Shishido , N. Kanehira , et al., “Molecular and Pathological Analyses of IARS1‐Deficient Mice: An IARS Disorder Model,” International Journal of Molecular Sciences 24 (2023): 6955, 10.3390/ijms24086955. 37108118 PMC10138339

[34] M. Boissan , U. Schlattner , and M. L. Lacombe , “The NDPK/NME Superfamily: State of the Art,” Laboratory Investigation 98 (2018): 164–174, 10.1038/labinvest.2017.137. 29451272

[35] T. Hirano , N. Kobayashi , T. Matsuhashi , et al., “Mapping and Exome Sequencing Identifies a Mutation in the IARS Gene as the Cause of Hereditary Perinatal Weak Calf Syndrome,” PLoS One 8 (2013): e64036, 10.1371/journal.pone.0064036. 23700453 PMC3660308

