# Supplementary material for: Atypical Presentation of IARS1 ‐Related Disorder: Expanding the Phenotype and Genotype
Source: JIMD Rep. 2025 May 12;66(3):e70020. doi: 10.1002/jmd2.70020 (PMC12069011; doi:10.1002/jmd2.70020)
Supplement: Supplementary file 1 — Data S1. Supporting Information. [file JMD2-66-e70020-s001.docx]

**Supplementary File**

Respiratory Chain Enzyme Analysis (Children’s Hospital Colorado, Aurora, CO)

| Enzyme activity (nmol/min.mg protein) | Proband | Control (Average) | Control (P5) | Control (P95) | Proband (% of average) | Z-score |
| --- | --- | --- | --- | --- | --- | --- |
| Complex I | 29.0 | 35.5 | 14.4 | 56.0 | 82% | -0.2 |
| Complex II | 218.3 | 232.5 | 174.7 | 309.8 | 94% | -0.1 |
| Complex III | 13.4 | 19.0 | 13.8 | 27.6 | 70% | -0.7 |
| Complex II+III | 19.0 | 58.9 | 10.8 | 107.3 | 32% | -1.3 |
| Complex IV | 1.4 | 1.8 | 0.5 | 3.2 | 81% | -0.2 |
| Citrate synthase | 86.3 | 84.4 | 59.5 | 109.3 | 102% | 0.2 |

| Ratio over citrate synthase | Proband | Control (Average) | Control (P5) | Control (P95) | Log ratio | Z-score |
| --- | --- | --- | --- | --- | --- | --- |
| Complex I | 336 | 441 | 162 | 730 | 0.76 | -0.3 |
| Complex II | 2530 | 2811 | 2304 | 3311 | 1.21 | -0.6 |
| Complex III | 155 | 237 | 128 | 315 | 0.58 | -0.8 |
| Complex II+III | 220 | 686 | 138 | 1062 | 0.66 | -1.5 |
| Complex IV | 17 | 22 | 6 | 335 | 0.08 | -0.2 |

| Ratio over Complex II | Proband | Control (Average) | Control (P5) | Control (P95) | Log ratio | Z-score |
| --- | --- | --- | --- | --- | --- | --- |
| Complex I | 133 | 156 | 68 | 252 | 0.63 | -0.2 |
| Complex III | 61 | 85 | 50 | 116 | 0.48 | -0.7 |
| Complex II+III | 87 | 246 | 62 | 383 | 0.55 | -1.4 |
| Complex IV | 7 | 8 | 3 | 13 | 0.07 | -0.1 |

Mitochondrial DNA Content Analysis (qPCR) Analysis (Baylor Genetics, Houston, TX)

| Specimens | Amount | % of Control |
| --- | --- | --- |
| Control (age 0-5y) | 2025-3646 (3134 ± 733) |  |
| Proband | 1035 | 33% |
| POLG (n = 1) |  | 3% |
| DGUOK (n = 5) |  | 3-7% |
| MPV17 (n = 5) |  | 2-18% |

**
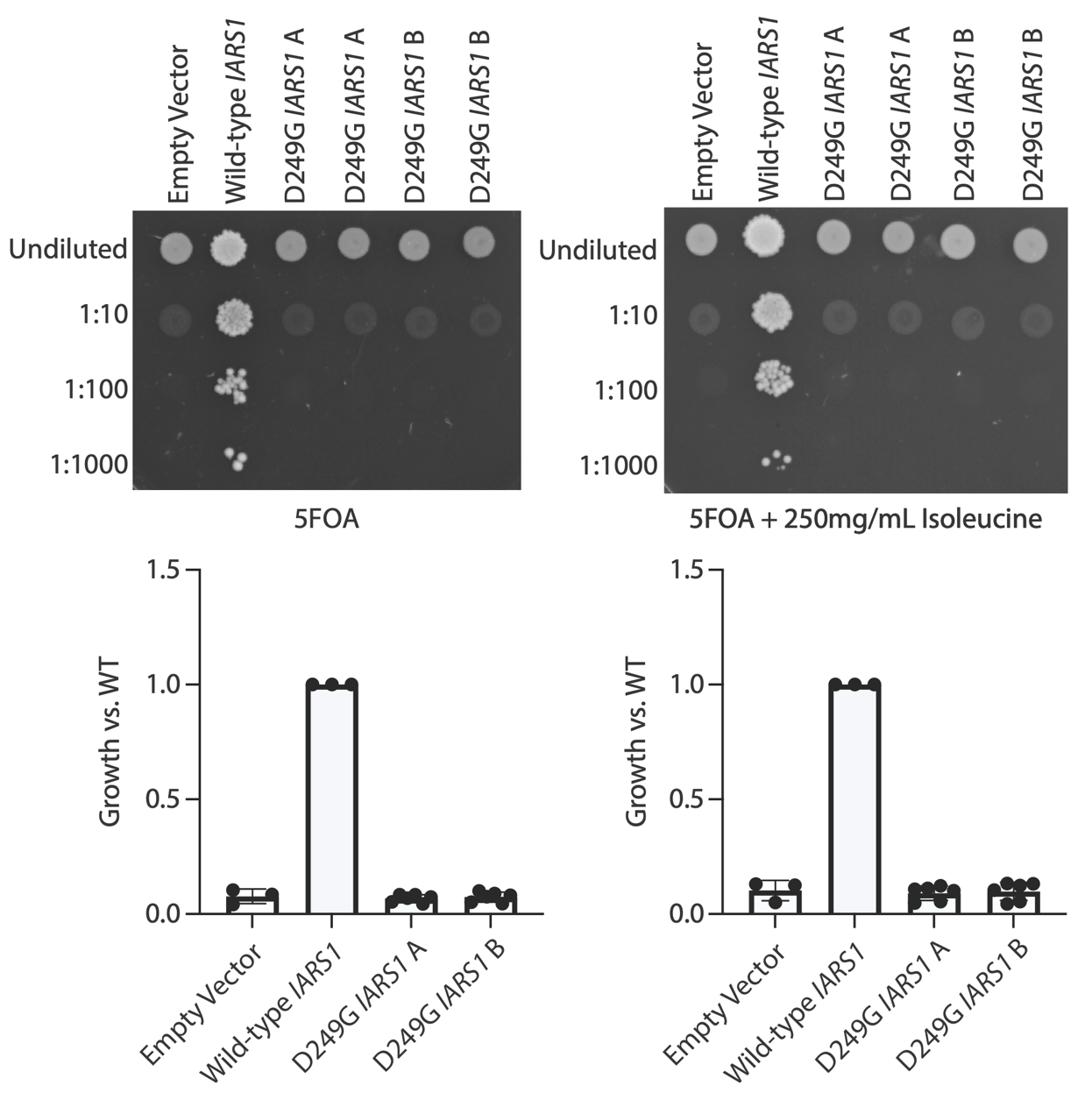
**

**Supplementary Figure 1. Attempts to improve p.Asp249Gly *IARS1* function with leucine supplementation**. Top panels: Yeast lacking endogenous *ILS* (the yeast ortholog of *IARS1*) and maintained by a *URA3*-bearing plasmid bearing wild-type *ILS* were transformed with plasmids containing wild-type IARS1, p.Asp249Gly *IARS1* (noted as‘D249G’), or no insert (‘Empty’). Resulting cultures were plated undiluted or diluted (1:10, 1:100, or 1:1,000) on media containing 5-FOA or containing 5-FOA and an additional 250mg/mL isoleucine. Two independently generated mutant constructs (‘A’ and ‘B’) were generated. Bottom panels: Quantification of yeast growth at day three across three independent trials. All data are normalized to growth associated with wild-type *IARS1*
